# The Complex Interaction between Proton Pump Inhibitors and Cancer Treatment

**DOI:** 10.3390/cancers15225346

**Published:** 2023-11-09

**Authors:** Hao Chen, Masaaki Kondo, Nobuyuki Horita, Kenichi Takahashi, Takeshi Kaneko

**Affiliations:** 1 Chemotherapy Center, Yokohama Minami Kyosai Hospital, Yokohama 236-0037, Japan; 2 Department of Pulmonology, Yokohama City University, Yokohama 236-0004, Japan; takeshi@yokohama-cu.ac.jp; 3 Chemotherapy Center, Yokohama City University Hospital, Yokohama 236-0004, Japan; 4 Department of Respiratory, Yokohama Minami Kyosai Hospital, Yokohama 236-0037, Japan

We have read the article authored by Rizzo et al., titled “Impact of Proton Pump Inhibitors and Histamine-2-Receptor Antagonists on Non-Small Cell Lung Cancer Immunotherapy: A Systematic Review and Meta-Analysis,” which was published in the journal *Cancers* [1]. Proton pump inhibitors (PPIs) and Histamine 2 receptor antagonists (H2RAs) have long played crucial roles in gastrointestinal therapy, particularly in the management of acid-related disorders such as gastroesophageal reflux disease (GERD) and peptic ulcers [2]. However, the interplay between PPIs and chemotherapy is multifaceted. PPIs not only have the potential to directly influence cancer but also exhibit various effects in combination with chemotherapy [3].

**Mechanism of PPIs**

PPIs operate by reducing stomach acid secretion. These drugs are absorbed in the proximal small bowel and subsequently affect the parietal cells of the stomach. The parietal cells contain the H^+^/K^+^ ATPase enzyme, known as the proton pump, which PPIs inhibit. This enzyme represents the final step in acid secretion in the stomach. Intriguingly, PPIs are considered prodrugs, becoming active only after undergoing acid-catalyzed cleavage within the acidic secretory canaliculi of the parietal cells. Hepatic P450 enzymes are responsible for metabolizing PPIs, with CYP2C19 playing a dominant role [4].

**The Dual Role of PPIs in Cancer Development**

PPIs exhibit the potential to promote tumor progression in several ways. Firstly, they induce an acidification effect within the tumor microenvironment, potentially creating a conducive milieu for cancer cells. An acidic microenvironment can hinder immune responses and facilitate tumor invasion and metastasis [5]. Secondly, PPIs may alter the gut microbiota, which can, in turn, influence cancer development. Dysbiosis can impact inflammation, immune responses, and the production of metabolites that either promote or inhibit cancer [6]. Thirdly, PPIs can lead to hypochlorhydria, characterized by low stomach acid levels, potentially affecting nutrient absorption and digestion, indirectly influencing cancer risk and progression, particularly in the case of gastric cancer [7]. Fourthly, PPIs could modify pH-dependent drug absorption, potentially reducing the effectiveness of certain tyrosine kinase inhibitors (TKIs) used in lung cancer treatment. By decreasing stomach acid, PPIs elevate the stomach’s pH, possibly diminishing the absorption of these TKIs and leading to suboptimal drug levels in the bloodstream [8].

Aside from promoting tumor progression, PPIs have displayed other potential mechanisms relevant to cancer treatment. Firstly, they exhibit anti-inflammatory effects by attenuating inflammation in the gastric mucosa through the inhibition of inflammatory cytokine production [9]. Since inflammation is a known driver of carcinogenesis, this property is especially relevant in cancer management. Secondly, PPIs possess anti-angiogenic effects, as some studies suggest they may inhibit angiogenesis—a critical process for tumor growth and metastasis—by modulating the expression of vascular endothelial growth factor (VEGF) and other angiogenic factors [10]. Thirdly, PPIs could induce apoptosis in cancer cells, potentially inhibiting tumor growth [11].

**PPIs and the Risk of Gastrointestinal Tumors**

The risk of gastrointestinal tumors caused by the long-term use of PPIs is a potentially important problem in clinical practice. Studies have indicated that the use of PPIs significantly increases the risk of gastric cancer, especially in Asian populations [12]. Long-term use of PPIs may heighten the risk of gastric cancer in individuals infected with Helicobacter pylori, a bacterium strongly associated with gastric cancer. PPIs can obscure the symptoms of gastritis, leading to delays in diagnosis and treatment. The type of esophageal cancer varies by region, with squamous cell carcinoma comprising more than 90% of cases in Asian countries like Japan, South Korea, and China, while adenocarcinoma accounts for 50 to 70% in Europe and the United States, with squamous cell carcinoma representing about 3% to 5%. PPIs do not appear to play a protective or risk-inducing role in esophageal adenocarcinoma [13], but they may have a preventive effect in esophageal squamous-cell carcinoma [14]. Moreover, PPI use has been associated with an increased risk of pancreatic cancer, liver cancer, and biliary tract cancer [15,16,17]. The relationship between PPI use and colorectal cancer remains controversial and not well established [17,18].

**Interaction of PPIs and Cancer Treatment**

PPIs can interact with certain chemotherapy drugs, affecting their absorption and efficacy. This interaction underscores the importance of personalized treatment plans and medication management in cancer therapy. In patients receiving treatment for breast cancer, both clinical progression-free survival (PFS) and overall survival (OS) were consistently poorer in those taking concomitant PPIs, whether they were receiving endocrine-sensitive or endocrine-resistant treatments [19]. However, in the treatment of esophageal squamous cell carcinoma, PPIs may enhance the effect of 5-FU [20]. PPIs can also amplify the effects of radiation therapy by reducing the acidity of tumor cells—a phenomenon known as radio-sensitization—which has shown promise in preclinical studies [21].

Immune checkpoint inhibitors (ICIs) are commonly used in cancer therapy. PPIs’ ability to reduce gastric acidity can impact the absorption of immunotherapeutic agents. Additionally, PPI-induced hypochlorhydria may affect the gut microbiota, potentially influencing the efficacy of immunotherapy. Several studies have reported worse outcomes with the combination of PPIs and ICIs in various cancers, including NSCLC, melanoma, urothelial carcinoma, renal cell carcinoma, and hepatocellular carcinoma [22]. This combination may also increase the risk of immune-related adverse events, particularly acute kidney injury.

EGFR-TKIs are increasingly used as first-line therapy for advanced NSCLC. PPIs or H2RAs with EGFR-TKIs have been associated with shorter PFS and OS and a higher risk of hepatotoxicity in NSCLC patients. The co-administration of PPIs or H2RAs should be avoided, but if necessary, H2RAs represent a preferable choice [23]. PPI use has been linked to both all-grade hepatotoxicity and grade 3–4 hepatotoxicity [24]. PPIs may also reduce the absorption of EGFR-TKIs, which are crucial in the treatment of various cancers. Some H2RAs, such as ranitidine and cimetidine, are CYP3A inhibitors, while most EGFR-TKIs are metabolized by CYP3A4. Consequently, the use of H2RAs could potentially increase the concentration of EGFR-TKIs, partially mitigating the influence of altered pH [25].

Nevertheless, integrating PPIs into cancer treatment is a complex endeavor. Clinicians must conduct a comprehensive risk-benefit assessment when prescribing PPIs to cancer patients, considering factors such as cancer type, stage, treatment plan, and individual patient characteristics. Regular monitoring of patients on chronic PPI therapy is essential to assess their response to treatment and to detect any adverse effects or drug interactions. Personalized treatment plans that account for the patient’s specific cancer and medical history should be developed, taking into consideration the potential impact of PPIs on treatment outcomes.

**Conclusions**

While originally designed for the management of acid-related disorders, PPIs are increasingly garnering attention as potential effects in cancer treatment. The connection between PPIs and cancer therapy is intricate and diverse, with the potential to both facilitate and impede cancer progression and its treatment. PPIs wield a substantial influence on the tumor microenvironment, gut microbiota, and drug interactions, all of which can exert a considerable impact on the outcomes of cancer therapy. Therefore, healthcare professionals must exercise thoughtful consideration of an individual patient’s medical history, the specific type of cancer they are dealing with, and the chosen treatment approach when contemplating the prescription of PPIs.
